# Biologically inspired robotic perception-action for soft fruit harvesting in vertical growing environments

**DOI:** 10.1007/s11119-023-10000-4

**Published:** 2023-03-13

**Authors:** Fuli Wang, Rodolfo Cuan Urquizo, Penelope Roberts, Vishwanathan Mohan, Chris Newenham, Andrey Ivanov, Robin Dowling

**Affiliations:** 1 School of Computer Science and Electronic Engineering, University of Essex, Colchester, CO4 3SQ UK; 2 Wilkin & Sons Ltd, Factory Hill, Tiptree, Essex CO5 0RF UK

**Keywords:** Soft fruit harvesting, Generative adversarial networks, Crop detection/localization, Dexterous manipulation

## Abstract

Multiple interlinked factors like demographics, migration patterns, and economics are presently leading to the critical shortage of labour available for low-skilled, physically demanding tasks like soft fruit harvesting. This paper presents a biomimetic robotic solution covering the full ‘Perception-Action’ loop targeting harvesting of strawberries in a state-of-the-art vertical growing environment. The novelty emerges from both dealing with crop/environment variance as well as configuring the robot action system to deal with a range of runtime task constraints. Unlike the commonly used deep neural networks, the proposed perception system uses conditional Generative Adversarial Networks to identify the ripe fruit using synthetic data. The network can effectively train the synthetic data using the image-to-image translation concept, thereby avoiding the tedious work of collecting and labelling the real dataset. Once the harvest-ready fruit is localised using point cloud data generated by a stereo camera, our platform’s action system can coordinate the arm to reach/cut the stem using the Passive Motion Paradigm framework inspired by studies on neural control of movement in the brain. Results from field trials for strawberry detection, reaching/cutting the stem of the fruit, and extension to analysing complex canopy structures/bimanual coordination (searching/picking) are presented. While this article focuses on strawberry harvesting, ongoing research towards adaptation of the architecture to other crops such as tomatoes and sweet peppers is briefly described.

**Supplementary Information:**

The online version contains supplementary material available at 10.1007/s11119-023-10000-4.

## Introduction

Precision agriculture brings a revolution to farming and food production, which will see the food we eat and how we produce it undergo a huge change (National Farmers Union, 2019). Meanwhile, the agricultural food industry is under severe pressure due to the critical shortage of labour available for tasks like fruit picking, packaging, increasing demand for produce, minimising production costs, and wastage and ensuring environmental sustainability. According to the Department for Environment Food & Rural Affairs statistics before the outbreak of the COVID 19 pandemic, the UK soft fruit market accounts for 18% of the annual levy income, and consumption of soft fruit increased by approximately 150% over the last five years. Strawberries have seen the biggest growth in the berry category. Additionally, the need is especially significant in the soft fruit sector, which uses 29,000 seasonal pickers to generate over 160,000 tons of fruit every year in the United Kingdom (British Summer Fruits, 2017). After Brexit, this labour force appears to be particularly insufficient. However, the UK is not alone, as the global population continues to grow, many countries urgently need to solve the problems of low production efficiency in fruit/vegetable production and efficient/intelligent utilisation of resources. Besides, present social distancing measures worldwide due to COVID 19 pandemic also imply that manual labours involved in picking and packaging tasks will not be able to work in close proximity to each other, further motivating the urgent need for robotic solutions in this domain.

### Deep learning approaches toward crop perception

In recent times, there have been several interesting approaches to tackle this challenge. Research on crop recognition technology is particularly extensive. A variety of recognition algorithms such as colour-based analysis, edge detection, *k*-means clustering, Bayes classifications and combinations of them have been provided and discussed; see (Jana et al., 2017; Zhao et al., 2016) and references therein. Although these simpler methods work with great performance for controlled conditions (Durand-Petiteville et al., 2017; Fadhel et al., 2018), they present difficulties when changed from the environment in which they were calibrated or need recalibration when the conditions vary. For this reason, deep learning-based object detection has recently been a research hotspot in agricultural robotics due to its powerful learning ability and advantages in dealing with natural occlusions, lightning variation, scale transformation, and background switches (Zhao et al., 2019). Therefore, many supervised neural networks have been introduced to detect fruit or vegetable for harvesting robots. For example, to improve machine vision performance in fruit detection for a strawberry harvesting robot, Yu et al. (2019) introduced the Mask Region Convolutional Neural Network (Mask-RCNN), which improved universality and robustness in a non-structural environment. Also, Ge et al. (2019) have made some improvements to a vision system to localise strawberries based on the Mask-RCNN. The method amid to avoid collisions between the gripper and fixed obstacles, but the localisation algorithm still needed to optimise and adapt to suit more complex situations, such as occluded and unusual hanging positions of the strawberries. Besides strawberries, Altaheri et al. (2019) created and tested a rich image dataset of date fruit bunches in an orchard that consists of more than 8,000 images of five date types in different pre-maturity and maturity stages. What’s more, a team at the University of Cambridge (Birrell et al., 2020) initially trained Vegebot to recognise the harvest-ready, immature, infected lettuce and background in the field by using the YOLOv3 (Redmon and Farhadi, 2018). Although the current deep learning models have a good performance in object detection, one of the major disadvantages is the need for a large dataset. This causes tedious work collecting and manual labelling of the data, a synthetic dataset is proposed as a solution to this problem.

By seeing the fruit segmentation process as an image-to-image translation problem, multiple features need to be considered, this paper presents a novel and flexible perception system based on a conditional Generative Adversarial Network which was trained using synthetic data.

### Robotic manipulation of crops for picking/harvesting

Besides crop recognition, some types of research also pay attention to the manipulation and end-effector in harvesting robots. For example, several control schemes of grippers for harvesting crops were designed in laboratory environments (Dimeas et al., 2015; Yaguchi et al., 2016; Zhang et al., 2020), but there were no field experiments to verify their performance on farms. Silwal et al. (2017) presented the design and field testing of a robotic system designed to harvest apples. The harvesting system successfully picked 127 of the 150 fruit attempted for an overall success rate of 84%. However, it is also necessary to ensure that crops are not damaged while improving the picking rate for more fragile soft fruits. The cherry harvesting robot developed in Japan consists of a 4-degree-of-freedom (DoF) manipulator, a 3D vision sensor and an end-effector (Tanigaki et al., 2008). Given the nature of the cherry tree, the team created an articulated manipulator with an axis that moves up and down and three axes that turn left and right, so the fruit can be harvested in any direction. However, experiments show that the manipulation action may damage the target fruit if other fruits besides it. Although the end-effector is equipped with soft rubber components, this does not always work. Once the soft fruit surface is slightly damaged, its preserved time will be greatly shortened.

Therefore, a critical challenge is to achieve soft manipulation with minimal contact with the soft fruit. A dual-arm robot was developed for harvesting tomatoes in a greenhouse (Ling et al., 2019). However, the DoF of this kind of double manipulator are limited; it has some restrictions under uncertain conditions. The Vegebot platform (Birrell et al., 2020) also developed a custom end effector and software to harvest iceberg lettuce, but it’s not yet suitable for commercialisation. Xiong et al. (2020) developed an autonomous strawberry-harvesting system, which installed a gripper at the end of the manipulator to pick strawberries. Still, the gripper was not dextrous enough and would contact the harvest-ready and immature strawberries simultaneously. Arad et al. (2020) developed a robot for harvesting sweet pepper fruit in a greenhouse to improve the performance in commercial greenhouses. However, the success rate of crop harvest still needs to be improved compared to human workers. Generally, to move the end-effector accurately towards the fruit, the inverse kinematics problem has to be computed. However, the same movement goal can be reached by an infinite number of combinations of the control variables. The optimal control theory (OCT), as a dominant theory of the classical engineering design technique, has been implemented in dedicated motion planning software, such as TRAC-IK (Beeson & Ames, 2015). From a mathematical perspective, the OCT can be expressed as: under the constraints of the equation of motion and the allowable control variables, the extreme value of the objective function (minimum value of the cost function) is obtained, which can be considered as an optimisation problem. A basic challenge within this approach is to derive the optimal control signal with non-linear time-varying systems, given a specific cost function and assumptions as to the structure of the noise (Mohan & Morasso, 2011). Additionally, getting stuck in local optimum has been a common problem in optimisation algorithms. An alternative to OCT, as a general theory of synergy formation, is the Passive Motion Paradigm (PMP: (Ivaldi et al., 1988)). To shift the cost function to the force field, this paper proposes a neural network implementation of the PMP for addressing motor control and synergy formation in agricultural robots.

### Commercially available systems

Besides the value of academic research, there are still some commercial prospects for these developments. At present, many companies are already developing and producing independent modular robots or other related technologies to provide agricultural services, such as OCTINION (Octinion is an innovative R&D company specialised in mechatronic product development applied to biological material) and THORVALD (Thorvald is committed to developing autonomous modular robots that can be configured for most agricultural environments). Also, a new robot being developed by Fieldwork Robotics, a spin-out company from Plymouth University, could let farmers pick more than 25,000 raspberries a day.

### Versatile, configurable ‘perception-action system’ for robotic harvesting- contributions of this article

Automation in AgriFood can be considered an extreme case with the critical challenge of dealing with a diverse range of produce, variations even in the produce of even the same type, changing environmental conditions, and manipulation tasks involved. While all exiting automation solutions presently available are finely tailored to the specific product, there exists tremendous scope for functional recycling of the underlying sensing/perception, manipulation and decision-making frameworks to bring in the much-needed ‘Versatility, Configurability, Modularity and Adaptivity’ in the automated harvesting/smart farming processes. This paper proposes a biologically inspired robotic perception-action system. Novelty in the proposed research can be summarised as follows:

1. *Compared with the existing detection methods, the proposed system uses conditional Generative Adversarial Networks (GAN) trained using synthetic data taking into account a range of environmental variances. This approach can be adapted to other crops and importantly eliminates the cumbersome manual data collection on farms and labelling of such data. The recognition/localization performance of the system is compared with human performance.*
2. *High variability in the canopy structure of the crop, occlusions, and minimizing damage due to contact impose a range of task-specific constraints for the robot action system. For the robot manipulation actions, this paper presents a novel neural control framework* (Mohan et al., 2018) *for goal-directed reaching taking into account a range of task constraints (gripper pose, joint limits, timing, bimanual coordination, alignment of the gripper/cutter to the stem). The action system is a forward/inverse model that can be used to simulate the consequences of actions for predictive planning as well as an extension to a range of tools coupled to the arm.*
3. *The perception-action system is implemented on the Essex Agricultural robot (a mobile vehicle with two arms, 3D printed Gripper/Cutters and a range of sensing capabilities). Field trials have been conducted with the robot in the Country’s first new vertical growing system for soft fruit at Tiptree, Essex within the framework of the Versatile project funded by Innovate UK.*

### The essex agricultural robot platform

**Fig. 1:**
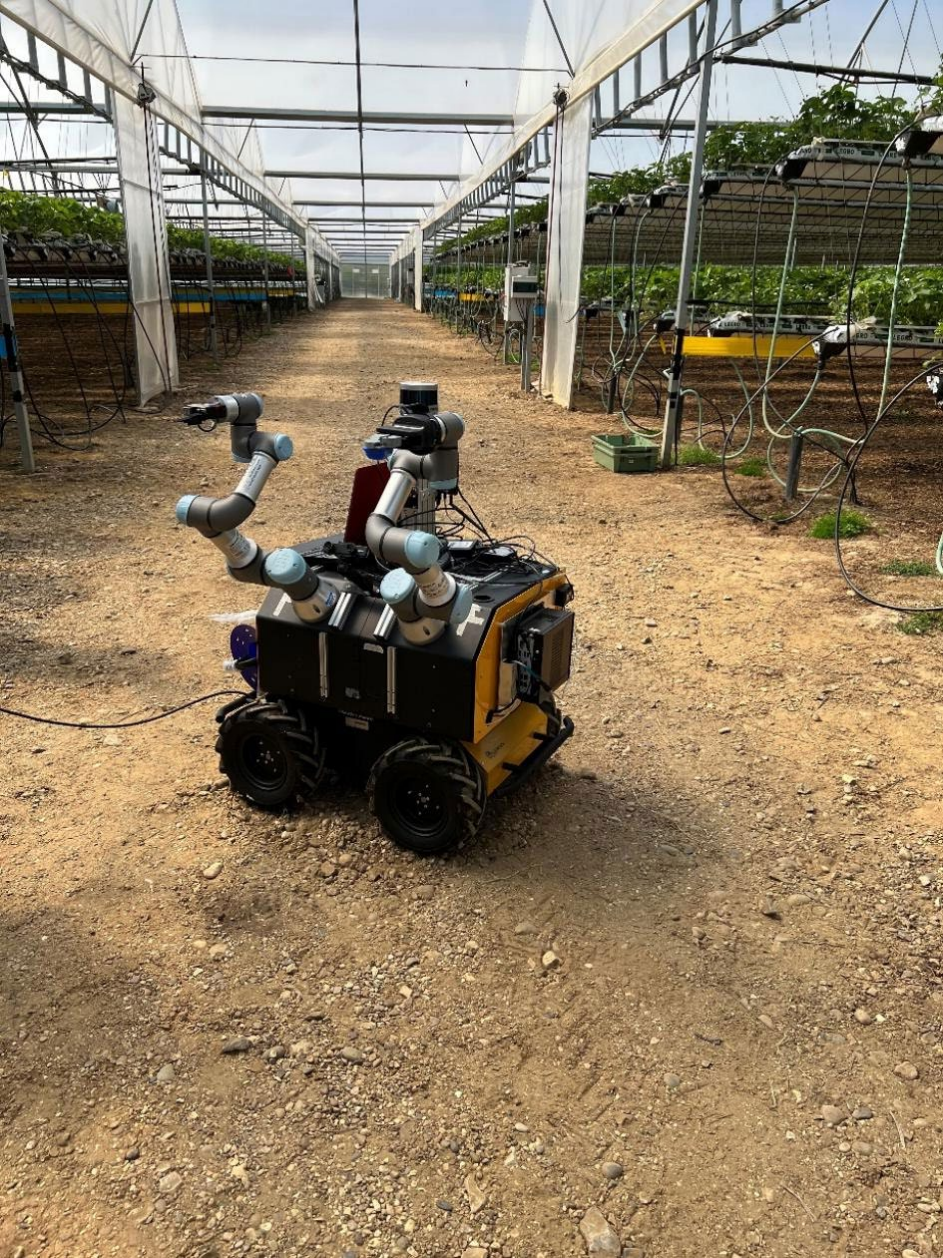
The Essex agricultural robot: a husky mobile robot with two 6-degree-of-freedom Universal Robots

As is shown in Fig. 1, the platform consists of a redesigned Husky unmanned ground vehicle (UGV) taking into account the dimensions of the vertical growing system, two UR3 robots (with all electronics and control rewired in the space underneath the mobile base and teach pendant replaced by a tablet). The arms can be coupled to a range of end-effectors based on the task like a multi-fingered/configurable soft hand, a 3D printed joint gripper/cutter designed in-house and a range of sensors for perception (stereo cameras, LIDAR). The design also takes into account modularity, reconfigurability enables the platform to be adaptable for a range of applications.

Compatibility of various hardware components (ex-soft hand with arm), ease of software integration using ROS and communication interfaces between hardware components, were given detailed consideration during the development of the robot.

## Perception system architecture

### Synthetic dataset

**Fig. 2:**
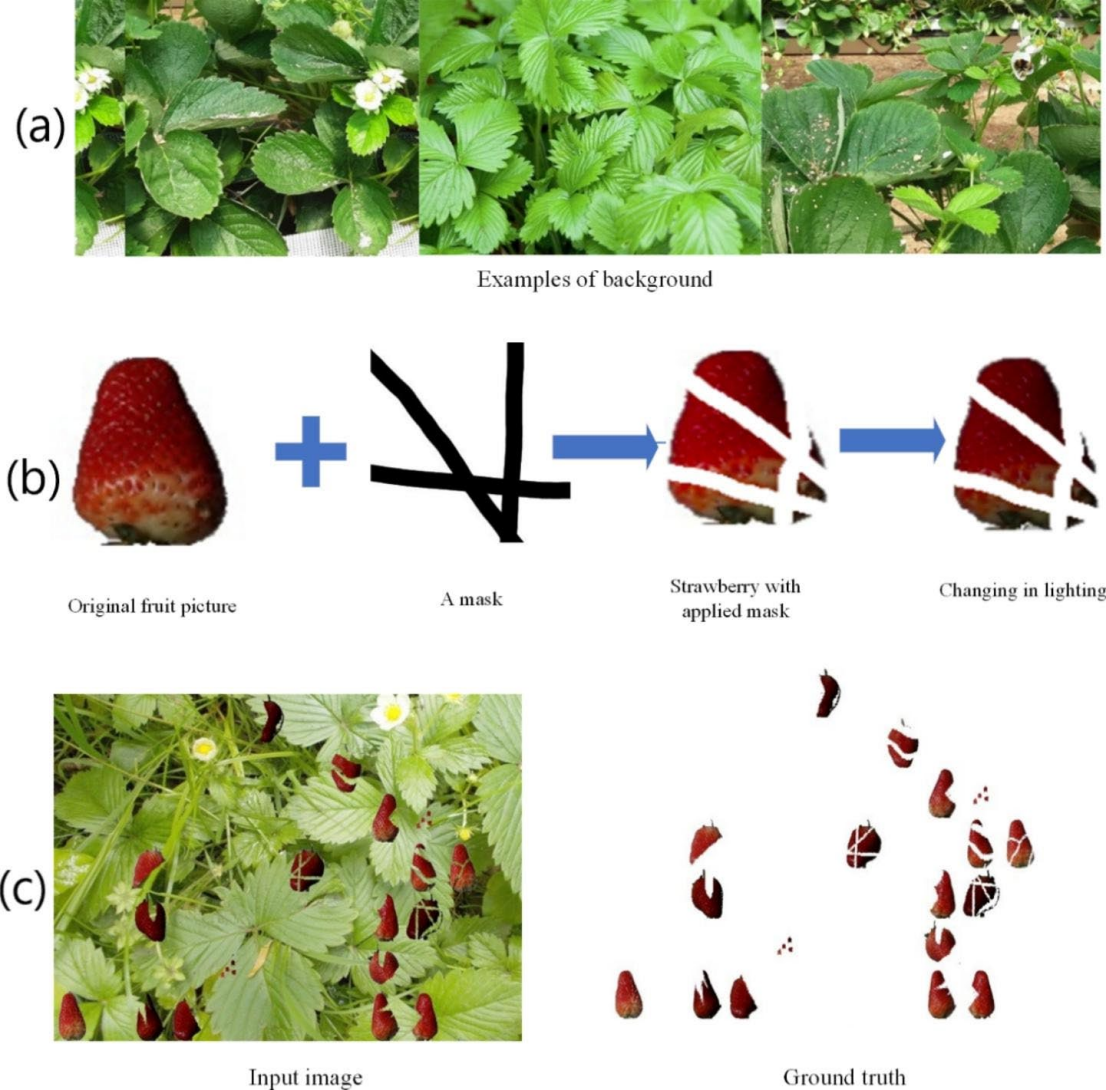
The generation process of training data set. (**a**): Some examples of background; (**b**) From the original fruit picture to the final image with mask and lighting changes applied; (**c**) A sample of the data set

While deep learning has played a pivotal role in the target recognition, data collection and labelling are time-consuming, especially when a complex environment and light conditions are required. The continuously changing travel restrictions due to COVID-19 also imply that a large amount of data from the field would be more troublesome. Using and creating a synthetic data set might be a solution to address the above concerns cause synthetic data have been used in some research (Barth et al., 2018; Rahnemoonfar & Sheppard, 2017). This paper also tried to generate the dataset by combining the fruit and background images. Specifically, a variety of background images were gathered from the internet and pictures taken from the farm. The pictures were chosen to be the most similar to the backgrounds and colours (green/brown) present in the field (see Fig. 2(a)). Then, the crops are needed to be placed on top of the background. We put pictures of individual strawberries from the biggest fruits dataset, namely, Fruits-360 (Mureşan & Oltean, 2018), on the background image to synthesise the data. Each strawberry is taken from its white background and lightning variation is accomplished by using gamma correction, a common non-linear operation for image illumination. Additionally, to create irregular crop images a bitwise-and operation is applied to the target crop image and a binary mask. The eleven masks used in this paper consisted of random lines and blobs emulating obstacles present in natural environments. The constructed dataset fits this objective by using strawberries from Fruit-360. In the end, the synthetic dataset contained 4,500 instances, with 900 instances for each fruit. The process can be visualised in Fig. 2(b). According to the synthetic process, the input image and ground truth for model training can be obtained simultaneously Fig. 2(c).

As a result, the advantage of this method is that the dataset required for training is automatically generated, with high efficiency and no manual labelling. The existing popular object detection models require people to customise their dataset, and labelling is time-consuming work. Using the current popular labelling processing software, people still need to deal with each image in front of the screen. Even if a picture takes a few seconds, the working time brought by thousands of pictures cannot be ignored. In this method, all training pictures can be automatically generated in a few minutes, and then network training can be started immediately.

### A perception system based on the GAN

In order to use this synthetic dataset to train the perception system model, we introduced the pix2pix model, one of the Conditional Generative Adversarial Networks to handle this work (Isola et al., 2017). The pix2pix model was designed to do the image-to-image translation, which can translate an input image into a corresponding output image by using the generator of a conditional GAN. According to our synthetic data, we introduced this translation idea into fruit detection; for example, in Fig. 2(c), the pix2pix model can translate the left image into the right image. Since we only need to identify maturate strawberries, no matter how complex the background environment is, this pix2pix model can make the complex environment simpler to detect the crop as we want. The original model worked with 256 × 256 images, and as the dimensions of the images increased, the model quality decreased. An improved model called Pix2pixHD (Wang et al., 2018) is introduced into our perception system to work with bigger images, so the generator of the model used is defined as follows: C64-C128-C256-R256-R256-R256-C256-C128-C64. C and R mean convolution and residual block respectively. Regarding the training process, the epoch was set as 300, batch size was 8.

**Fig. 3:**
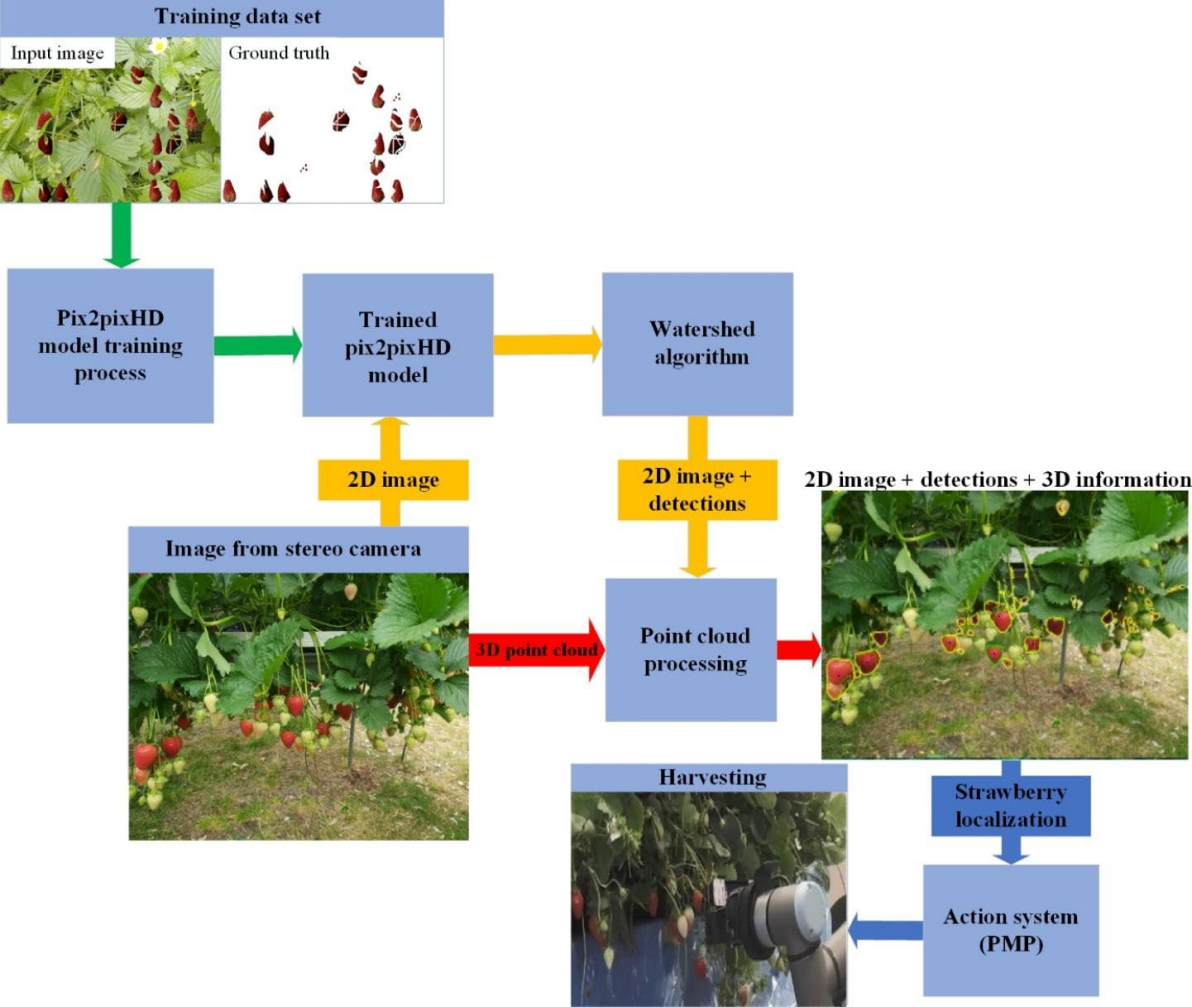
The overall architecture of the perception system: The green arrows show the process of image training; the yellow arrows indicate the target detection process; the red arrows indicate the acquisition of 3D information of the target; the blue arrows show the activation of the action system

After the translation work, we use the watershed algorithm (Puttemans et al., 2017) to estimate and divide the strawberries’ number (More details will be discussed in the section on performance analysis). The overall architecture of the proposed perception system is shown in Fig. 3. As is shown below, the Pix2pixHD model receives the 2D image from the stereo camera and inputs the translated image to the watershed algorithm for crop detection. Then, the camera combines the 2D information and accesses the 3D point cloud to localise the crops.

**Fig. 4:**
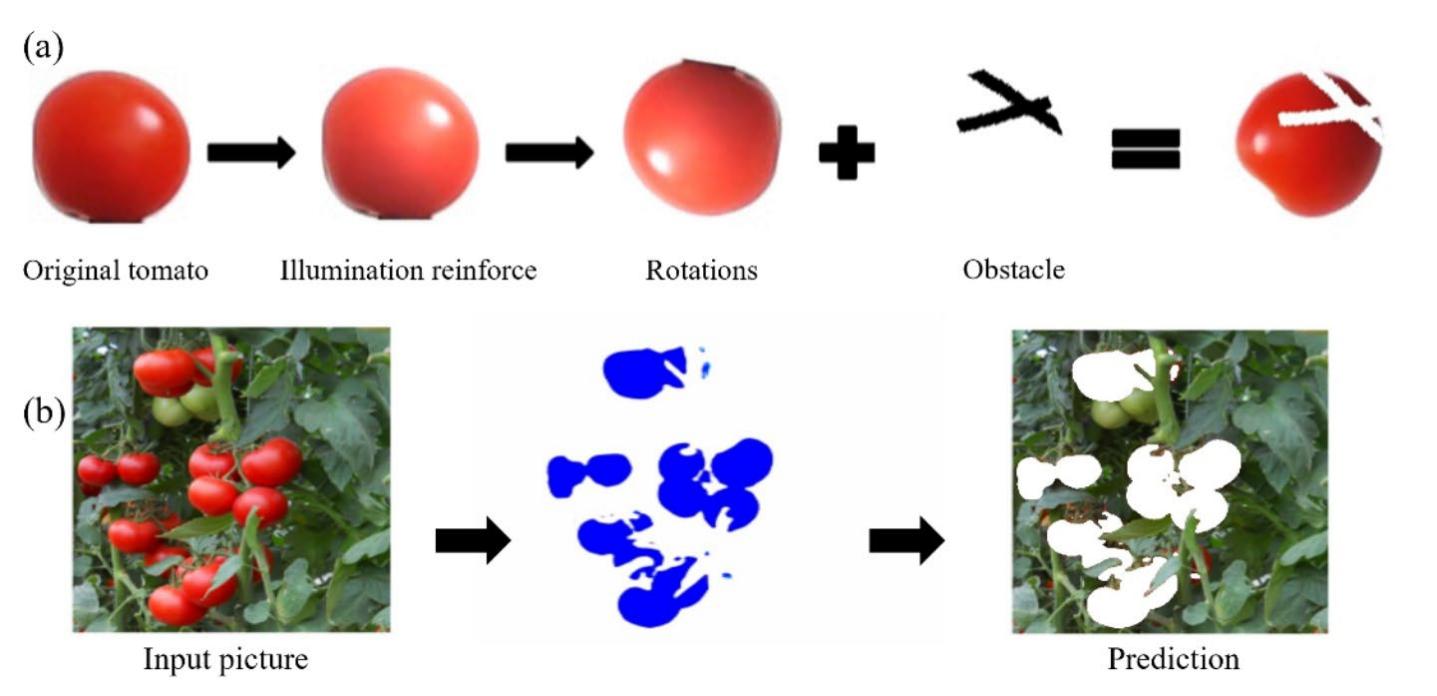
(**a**): In the process of data synthesis, replace the strawberry with tomato; (**b**) an example of tomato model predictions

For the proposed system, it can be extended to harvest other crops by changing the synthetic dataset and end-effector. As is shown in Fig. 4, the strawberry dataset was changed to tomato for training a new perception model. Besides, all generated dataset contains crops with different illumination, rotations and obstacles. Note that this paper focus on the strawberry application, more details about the performance of the model will be introduced in Sect. 5.

## The harvesting action generation system based on the passive motion paradigm

### Passive motion paradigm for goal-directed reaching

**Fig. 5:**
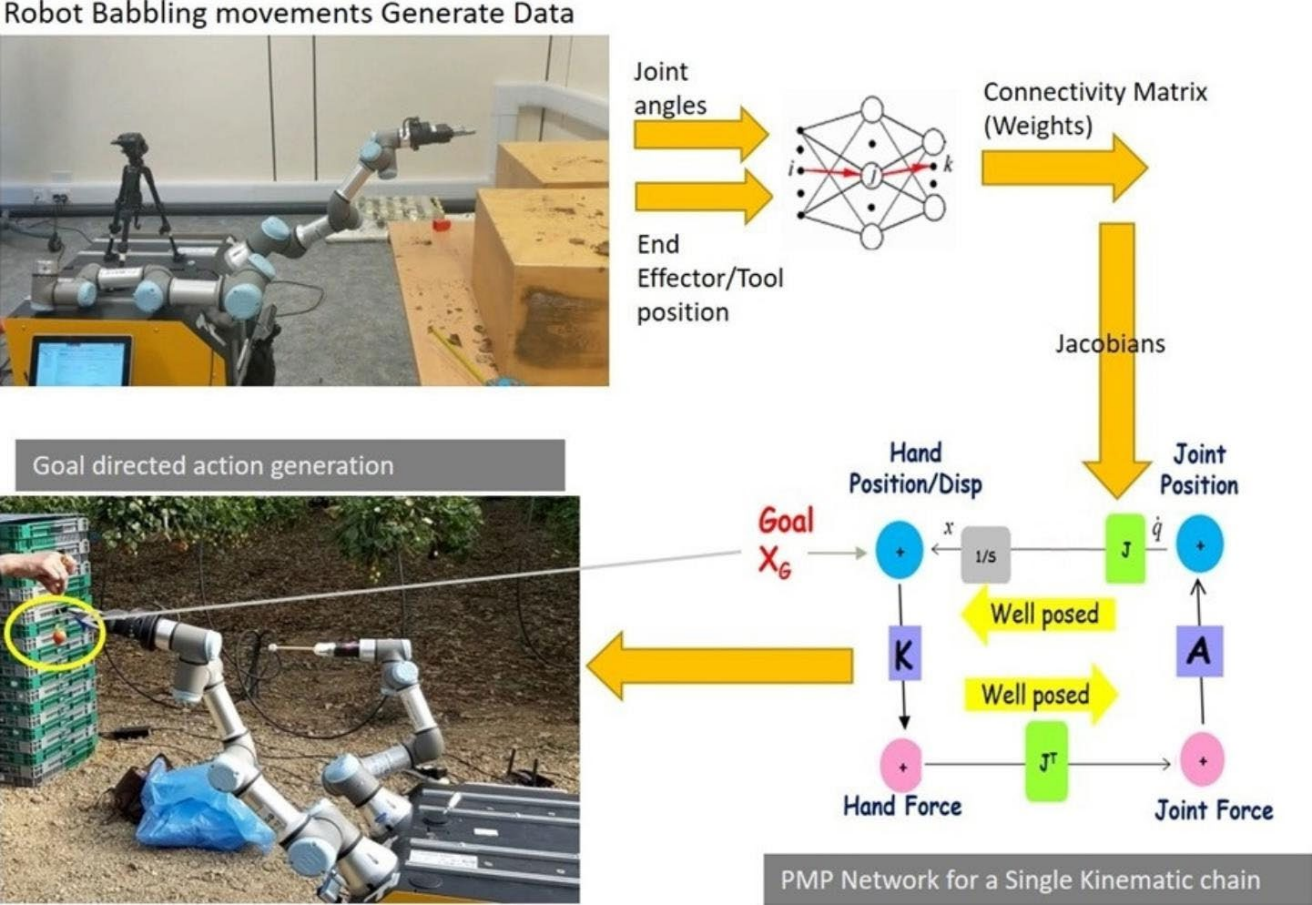
ANN based controller starts with babbling movements of the robot to generate data (top left) which is used to train the backpropagation network (top left). From the connectivity matrix, the Jacobians can be computed (bottom right and Eq. 2). The bottom left picture shows the arm reaching the target (X_G_).

The action system takes localisation information of the strawberry obtained from the point cloud and coordinates the two arms and the gripper/cutter of the robot for picking and other tool use actions required in the harvesting process. The action system developed for the robot is a neural network implementation of the Passive Motion Paradigm (Mohan et al., 2018; Mohan & Morasso, 2011) based on impedance control (Hogan, 1985), equilibrium point hypothesis (Bizzi et al., 1976, 1992). The architecture particularly enables (a) swift learning of the internal model of the arm/body and extension to the range of coupled tools; (b) runtime incorporation of a range of task constraints (end-effector pose, joint limits, tool orientation, motion trajectory and approach towards the target); (c) temporal synchronization and bimanual coordination for picking with two hands; (d) Forward Simulation of the consequences of action to support goal-directed reasoning. Figure 5 shows the block diagram summarizing the design of the ANN-based controller starting from data generation to goal-directed reaching with the robot. The steps are summarized below.

- *Data generation through robot babbling movements*. The training data for the ANN was obtained through the process of sensorimotor exploration/babbling. This is where, in the arms workspace, the UR3’s joint rotation readings and set of corresponding end-effector coordinates are saved into two files. The training set consists of 10,000 points in the workspace of the arm and corresponding joint angles.
- *Design of the neural controller*. Once the training data is obtained, a standard backpropagation network with two hidden layers was used to learn the mapping ${\mathbf{X}}=f\left( Q \right)$. In this case, ${\mathbf{Q}}=\left\{ {{q_i}} \right\}$ is the input vector (of joint angles of the UR3 arm), ${\mathbf{X}}=\left\{ {{x_k}} \right\}$ is the output vector (representing 3D position/orientation of the end-effector) ${\mathbf{Z}}=\left\{ {{z_j}} \right\}$ and ${\mathbf{Y}}=\left\{ {{y_l}} \right\}$ vectors are the output of the first and second hidden layer units of the neural network respectively. Equation 1 expresses the mapping, where $\left\{ {{\omega_{ij}}} \right\}$ are connection weights from the input layer to the first hidden layer, $\left\{ {{o_{jl}}} \right\}$ are the connection weights between two hidden layers, ${\mathbf{W}}=\left\{ {{w_{lk}}} \right\}$ are the connection weights from the second hidden layer to the output layer, ${\mathbf{H}}=\left\{ {{h_j}} \right\}$ are the net inputs to the neurons of the first hidden layer and ${\mathbf{P}}=\left\{ {{p_l}} \right\}$ are net inputs to the second hidden layer. Neurons of the two hidden layers fire using the hyperbolic tangent function; the output layer neurons are linear.

1 $${\mathbf{X}}=f\left( {\mathbf{Q}} \right) \Rightarrow \left\{ \begin{gathered} {h_j}=\sum\nolimits_{i} {{\omega _{ij}}{q_i}} \hfill \\ {z_j}=g\left( {{h_j}} \right) \hfill \\ {p_l}=\sum\nolimits_{j} {{o_{jl}}{z_j}} \hfill \\ {y_l}=g\left( {{p_l}} \right) \hfill \\ {x_k}=\sum\nolimits_{l} {{w_{lk}}{y_l}=\sum\nolimits_{l} {{w_{lk}} \cdot g\left( {\sum\nolimits_{j} {{o_{jl}}{z_j}} } \right)} } \hfill \\ \Rightarrow {x_k}=\sum\nolimits_{l} {{w_{lk}} \cdot g\left( {\sum\nolimits_{j} {{o_{jl}} \cdot g\left( {\sum\nolimits_{i} {{\omega _{ij}}{q_i}} } \right)} } \right)} \hfill \\ \end{gathered} \right.$$

Concerning the use of external objects as tools, the same procedure can be applied with the data (end-effector motion and the corresponding consequence on the tool effector) acquired also by imitating the teacher’s demonstration (Mohan et al., 2011; Mohan & Morasso, 2012) thus constraining the domain of random exploration.

From the learning weights of the neural network, it is possible to extract the Jacobians encoding the geometric relationship between the respective motor spaces (joint space-end effector space of the UR3 arm) using the chain rule (Eq. 2).

2 $$J=\frac{{\delta {x_k}}}{{\delta {q_i}}}=\sum\nolimits_{l} {{w_{lk}} \cdot {g'}\left( {{p_l}} \right)\sum\nolimits_{j} {{o_{jl}} \cdot {g'}\left( {{h_j}} \right){\omega _{ij}}} }$$

**Fig. 6:**
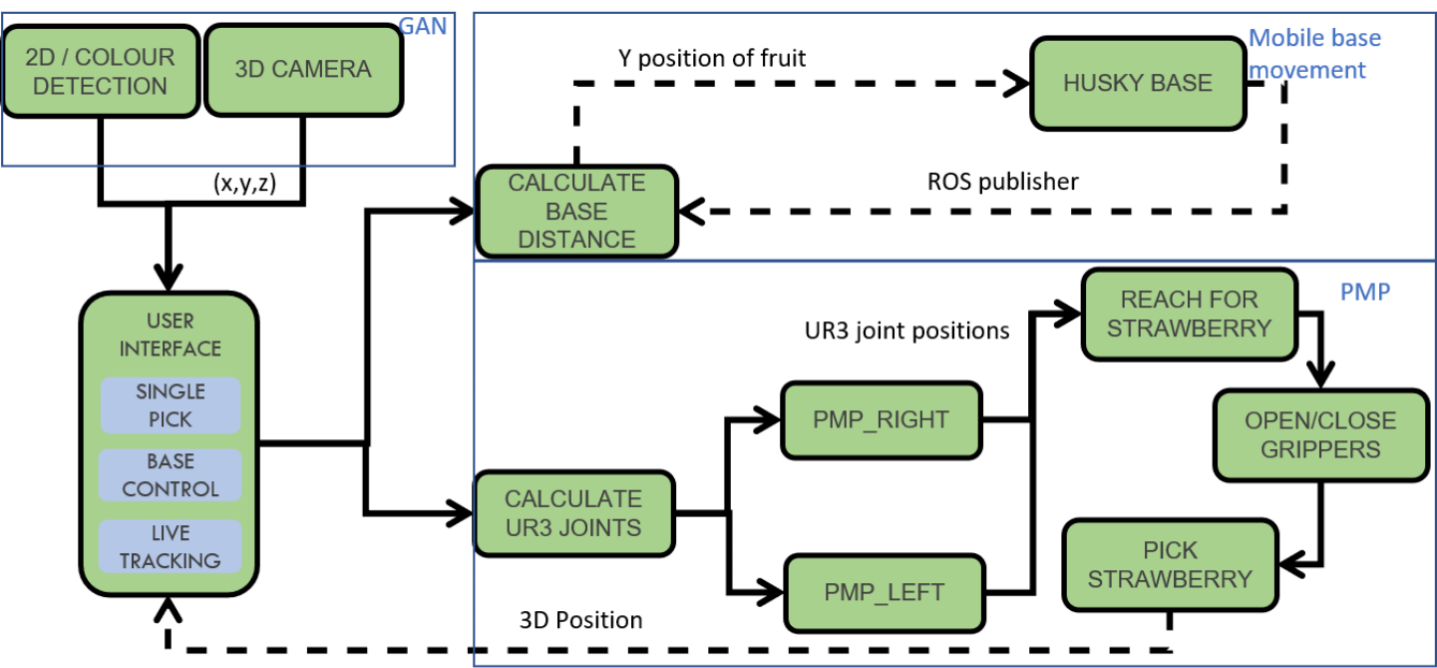
Integrated Perception-Action system- Core Building blocks

**Fig. 7:**
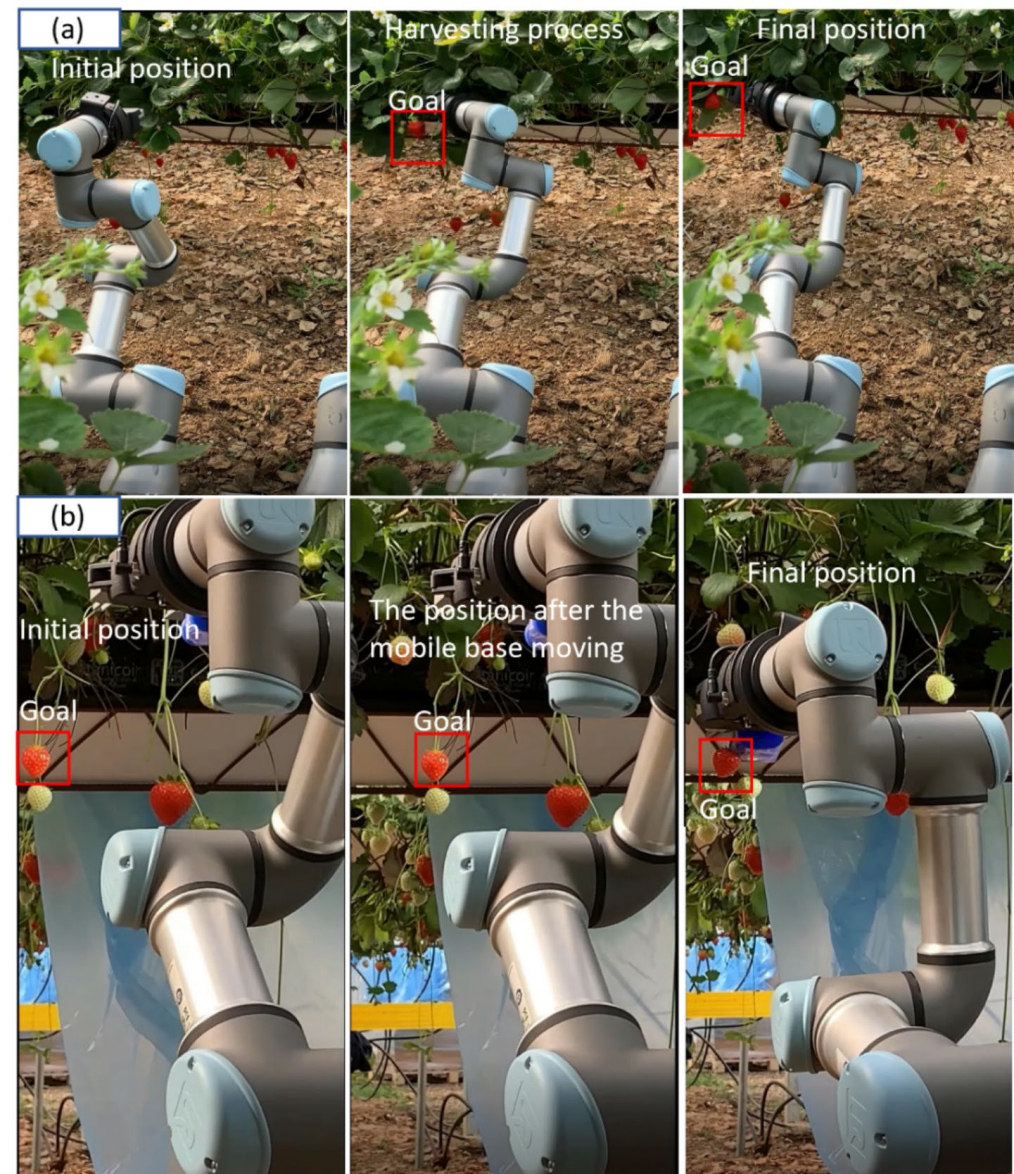
(**a**) Reaching a target without the mobile base movement; (**b**) reaching a target with the mobile base movement

**Fig. 8:**
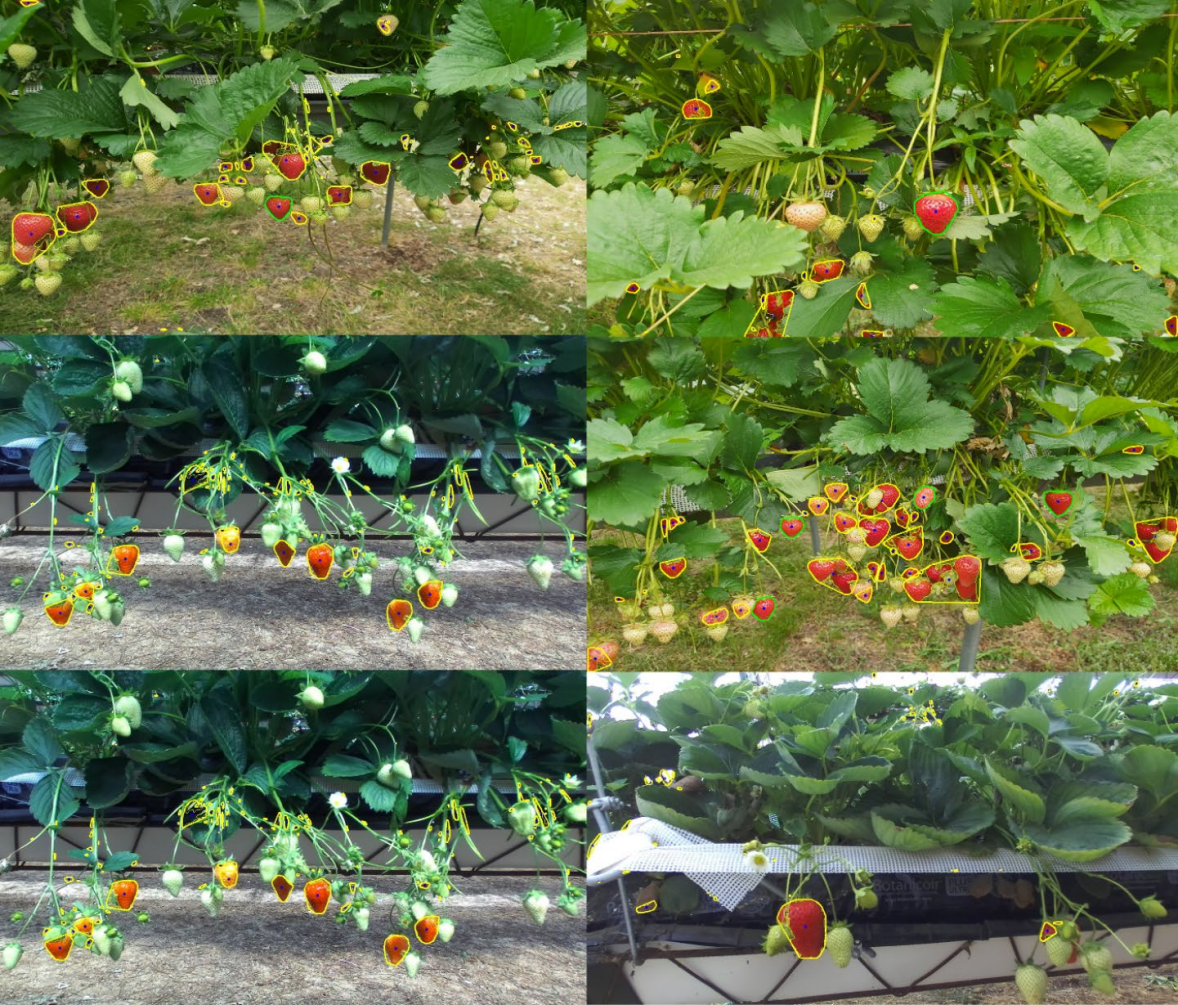
Strawberry detection and localisation in natural conditions

**Fig. 9:**
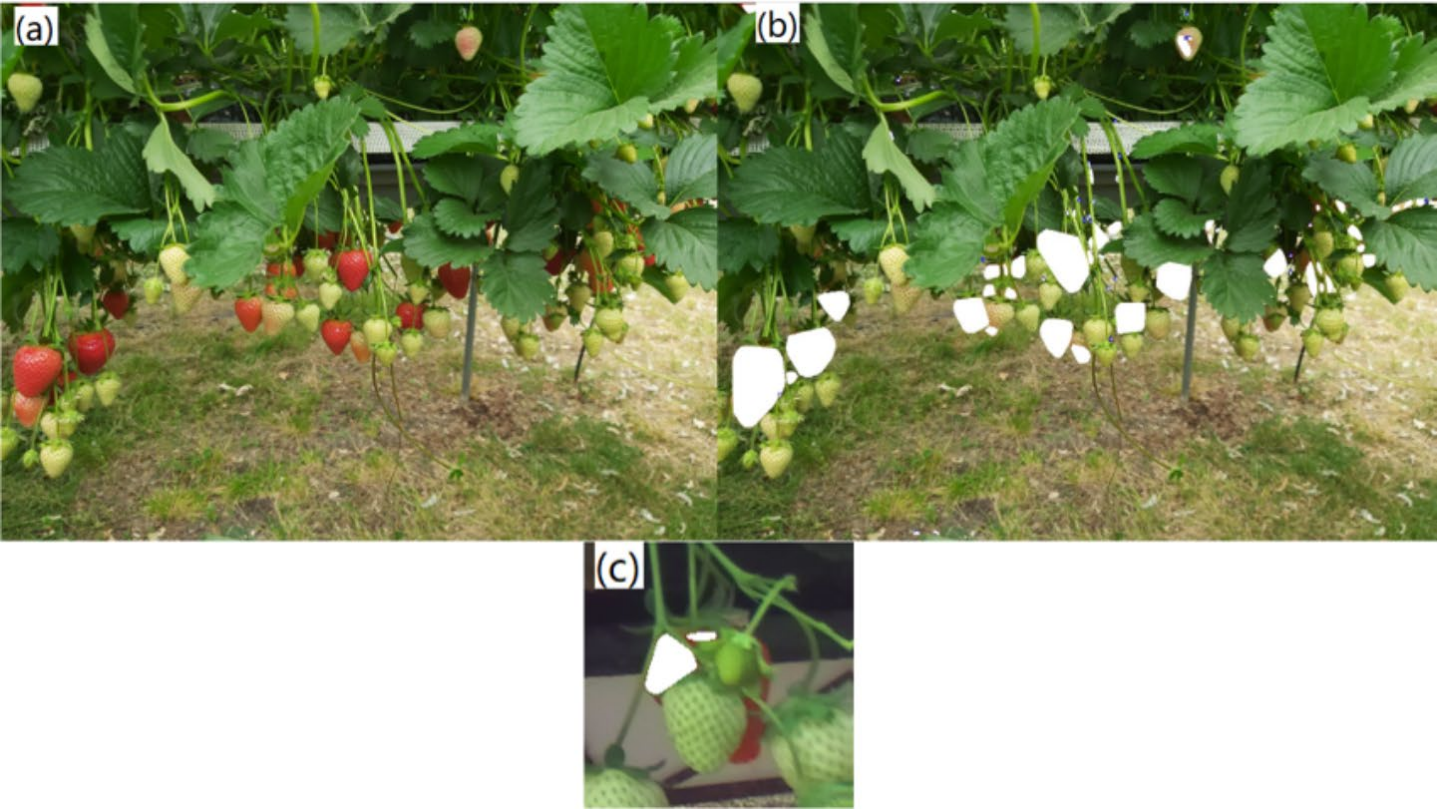
Performance measurement example. (**a**): Original image; (**b**): Remaining undetected sections after recognition; (**c**): Partially detected strawberry

**Fig. 10:**
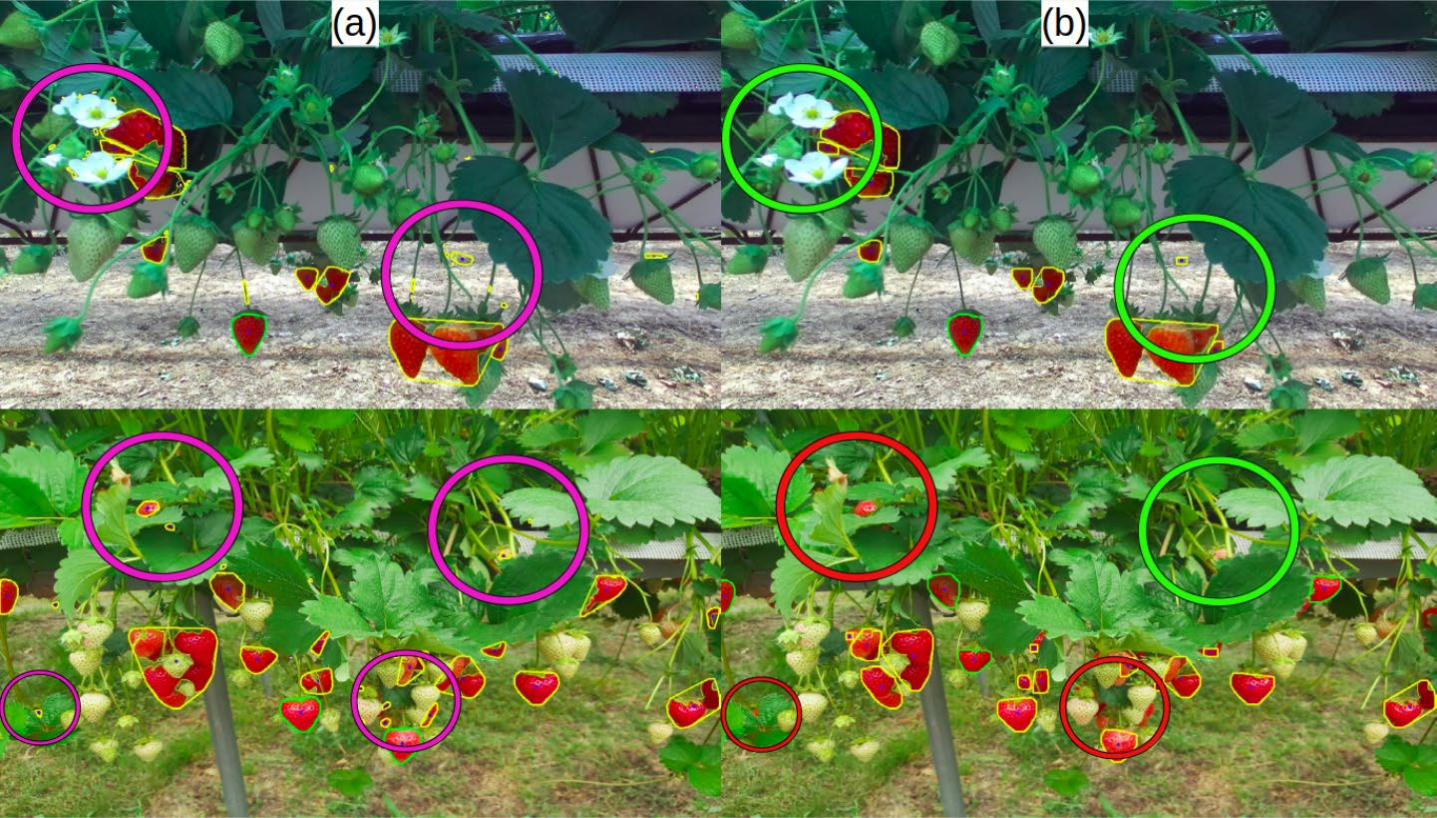
Morphological Operations: (**a**) predictions without operations applied (**b**) predictions with operations applied. Purple circles point out areas with small blobs. The noises are eliminated in green circles. Some correctly localised crops are in red circles

**Fig. 11:**
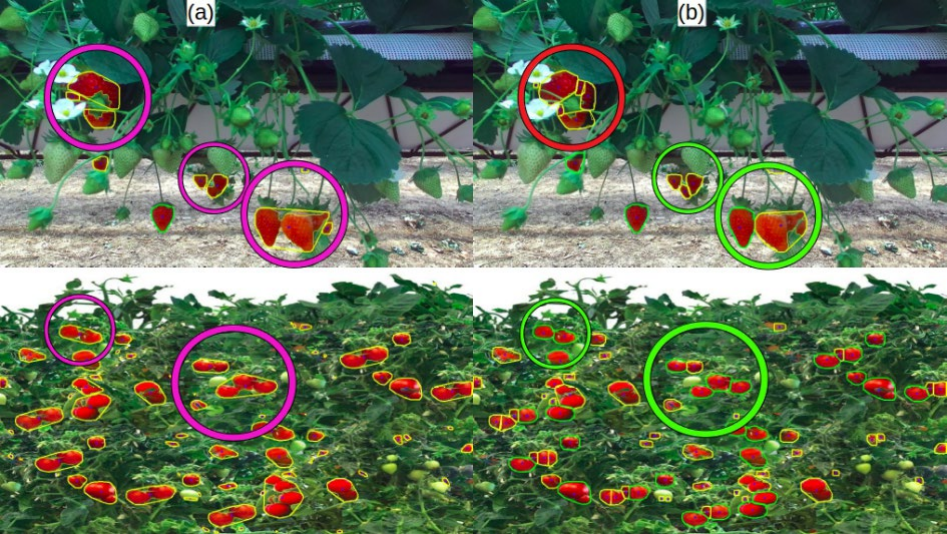
Applied Watershed algorithm to blobs with an area larger than 3000 pixels. (**a**) predictions without watershed algorithm (**b**)predictions with the watershed algorithm. Purple circles point out blobs that the watershed method will be applied; Green circles where the blobs cluster was correctly divided, and red ones when they were not

**Fig. 12:**
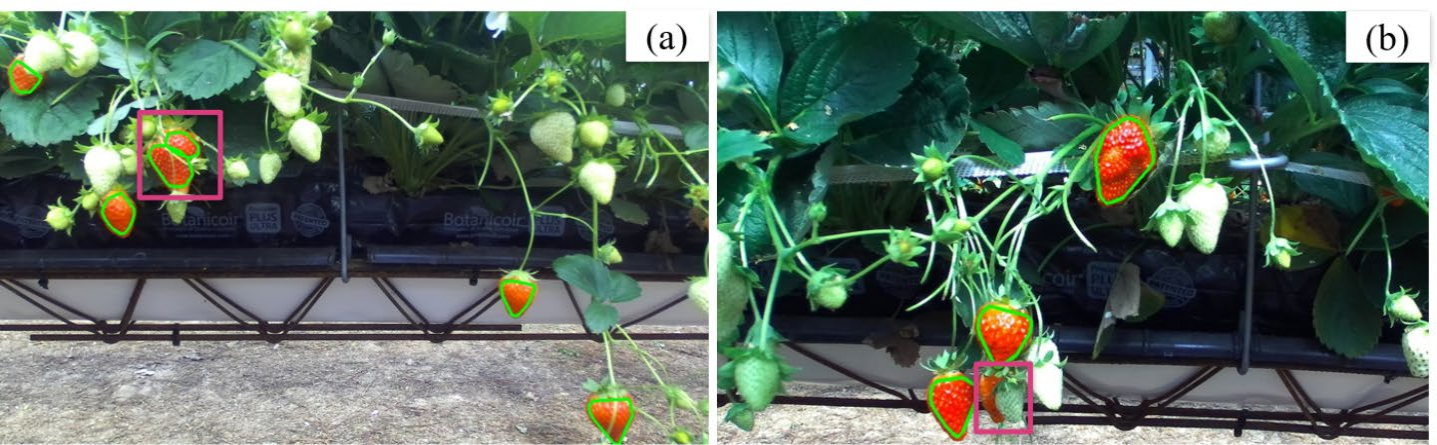
The situations where the perception system cannot count strawberries accurately. (**a**) wrong segmentation; (**b**) overlapping

**Fig. 13:**
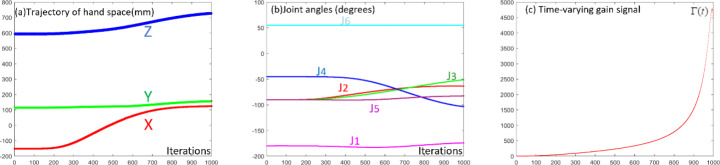
**a**) Sequence of end effector position from an initial position (-151, 116, 593) to the target (124, 158, 727) as a function of time; **b**) sequence of joint angles in all the DoF of the arm from an initial state to the final state (when the end effector reaches the goal); (**c**) Time-varying gain signal

c) *PMP network and Goal-directed reaching.* Once the ANN is trained, the PMP network can be generated for goal-directed reaching/control of the arm. The network shown in Fig. 5 represents the kinematic chain of a single arm. In this case, there are two motor spaces i.e. hand space with two nodes: representing force (pink) and position of the hand (blue) and arm joint space with two nodes representing torque (pink) and rotation of the various joints (blue). We call the pair of force-displacement nodes as a *work-unit (WU)*, because the scalar work ($force \times displacement$) is the structural invariant across different motor spaces. The network can be animated by attaching force fields to one or more body parts/ effectors in a goal-oriented fashion. The animation process is analogous to the coordination of a marionette with attached strings (that represent the attractor dynamics of the force field induced by the intended goal i.e. the strawberry). While reaching is the simplest case with a fixed point attractor (at the target), the body schema can be animated with moving point attractors to produce diverse spatiotemporal trajectories, as shown in the case of drawing (Mohan et al., 2011), tool use (Bhat & Mohan, 2015; Mohan & Morasso, 2012). The computational model can be summarized as follows (Figs. 6, 7, 8, 9, 10, 11, 12, 13).

Let ${\mathbf{q}}$ be the set of all the degrees of freedom (DoFs) that characterize the UR3 arm. Then the kinematic transformation ${\mathbf{x}}=f\left( {\mathbf{q}} \right)$ can be expressed as: ${\mathbf{\dot {x}}}=J \cdot {\mathbf{\dot {q}}}$ where *J* is the Jacobian matrix of the transformation extracted from the trained ANN. Next, the PMP animation in the simplest case for a serial kinematic chain involves the following steps:

1. *Generate a target-centred, virtual force field in the extrinsic space*:

3 $${\mathbf{F}}={K_{ext}}({{\mathbf{x}}_G} - {\mathbf{x}})$$

Where ${{\mathbf{x}}_G}$ is the strawberry to reach and ${K_{ext}}$ the virtual stiffness of the attractive field in the extrinsic space. ${K_{ext}}$ determines the shape and intensity of the force field. In the simplest case, *K* is proportional to an identity matrix and this corresponds to an isotropic field, converging to the target along straight flowlines.

(2) *Map the force field from the extrinsic space into the virtual torque field in the intrinsic space*:

4 $${\mathbf{T}}={J^T}{\mathbf{F}}$$

(3) *Relax the arm configuration to the applied field*:

5 $${\mathbf{\dot {q}}}={A_{\operatorname{int} }} \cdot {\mathbf{T}}$$

Where ${A_{\operatorname{int} }}$ is the virtual admittance matrix in the intrinsic space: the modulation of this matrix affects the relative contributions of the different joints to the overall reaching movement.

(4) *Map the arm movement into the extrinsic workspace*:

6 $${\mathbf{\dot {x}}}=J \cdot {\mathbf{\dot {q}}}$$

(5) *Integrate over time until equilibrium*:

7 $${\mathbf{x}}\left( t \right)=\int_{{{t_0}}}^{t} {J{\mathbf{\dot {q}}}d\tau }$$

The fifth step is integration, which gives us a trajectory with the equilibrium configuration ${\mathbf{x}}\left( t \right)$ defining the final position of the robot in the extrinsic space. Note that all the computations in the above loop are “well-posed” and the relaxation mechanism does not require any cost function to be specified to solve the indeterminacy related to the excess DOFs (the redundancy problem). A way to explicitly control time is to insert in the non-linear dynamics of the relaxation process (Eqs. 3–6), a time-varying gain $\varGamma \left(t\right)$ according to the technique originally proposed by (Zak, 1988) for content addressable memories and extended in the context of goal-directed reaching for robots (Bhat et al., 2017).

This can be implemented by substituting the relaxation Eq. (5) with the following one:

8 $${\mathbf{\dot {q}}}=\Gamma (t) \cdot {A_{\operatorname{int} }} \cdot {\mathbf{T}}$$

where a possible form of time-varying gain is the following that uses a minimum-jerk generator with duration *t* ):

9 $$\Gamma (t)=\frac{{\dot {\xi }}}{{1 - \xi }}$$

Where

10 $$\xi (t)=6{(t/\tau )^5} - 15{(t/\tau )^4}+10{(t/\tau )^3}$$

In general, a TBG can also be used as a computational tool for synchronizing multiple relaxations in composite PMP networks, coordinating the relaxation of movements of two arms or even the movements of two robots.

In the case of a simple reaching task with an arm, at the end of the animation process, we get four sets of trajectories as a function of time (shown in Fig. 13): (1) Sequence of joint angles given by the position node in the joint space (arm); (2) The resulting consequence i.e. the sequence of end-effector position given by the position node in end-effector space; (3) The sequence of torques at the different joints (arm and waist), given by the force node in the joint space; (4) The resulting consequence i.e. the sequence of forces applied by the end effector given by the force node in the end-effector space. The time-varying gain signal is shown in Fig. 13c which ensures that the target is reached in 1000 iterations. The time-varying gain can be thought as a temporal pressure that becomes stronger and stronger as the deadline approaches and diverges afterwards. Further details of the mathematical model for terminal attractor dynamics applied to goal-directed reaching in robots can be found in (Bhat et al., 2017).

At the same time, it is possible to integrate a range of internal and external constraints at runtime based on the requirements of the task that needs to be performed, in the form of force fields defined either in the extrinsic space or in the intrinsic space. The rest of the paper presents the results of both the perception system, action system and the integrated framework during field trials.

### Integrated perception-action software architecture

To realise the strawberries harvesting in the greenhouse, the proposed perception and action system are integrated into the Essex agricultural robot. The overall block diagram of the execution process of the robot is shown in Fig. 6. This whole system is designed to combine body and arm movement for goal-directed reaching. Specifically, as is shown in Fig. 7(a), if the system only coordinates the arm without the UGV/mobile base movement, although the target is reachable, the arm sometimes can reach a target at awkward angles. However, when the mobile base movement is combined, it can guarantee the target is always in front of the arm by adjusting the UGV. Then the arm can reach the target smoothly and the gripper can cut the stem in the horizontal direction. (see Fig. 7(b)). The detailed performance analysis of the system is as follows.

### Analysis of proposed perception system

The proposed perception system comprises both identification (detect the maturate strawberries) and localisation (see (Ortiz et al., 2018) for more details of the point cloud generated by the camera). To test the perception system’s validity and performance, we collected some real images from a strawberry greenhouse to test proposed perception system. Firstly, a number of images were selected to test the model containing different conditions and multiple strawberries (6 example images are shown in Fig. 8).

The results presented are without the use of the watershed algorithm nor the morphological operations, which will be discussed later. After the detection, the regions were cropped out from the original image (Fig. 9(a)) and then the remaining undetected sections or complete ripe strawberries would be analysed (Fig. 9(b)). If a strawberry is partially detected, then the undetected section is not counted (Fig. 9(c)). This is because the robot is expected to explore that area with the information of the detected portion and better detect the whole target. With this method, it is easy to detect any crops not detected by the system visually. Using this testing condition and measurements, 100% of ripe strawberries in the images selected can be detected. However, the system presents 81.4 blobs per image, and each image has two up to 30 visible strawberries.

Further, it is worth mentioning that there are two important operations to eliminate small blobs (noise) and segment very close strawberries in the perception system. The first operation is a morphological operation that is used for eliminating noise. Besides, the watershed algorithm allows for counting the objects or for further analysis of the separated objects (see (Kornilov & Safonov, 2018) for the algorithm implementations in open source libraries). The application comparison results of these two operations are shown in Figs. 10 and 11, respectively. Although the two operations can improve the performance of the perception system, they cannot guarantee all ripe strawberries can be accurately divided. To more specifically analyse this performance, 50 images were captured from the farm to estimate the error rate in the number of strawberries. First, the perception system was used to detect and count ripe strawberries in each image and then compared with the manual counting. The following equation was used to estimate the error rate in the number of strawberries.

11 $$Error=\frac{{\left| {nu{m_m} - nu{m_p}} \right|}}{{nu{m_m}}}$$

Where, $nu{m_m}$ is the number of ripe strawberries counted manually. $nu{m_p}$ is the perception system output. For all fifty testing images, Eq. (11) was used to estimate the error rate of each image, and then the average error rate was calculated as 10.83%. As is shown in Fig. 12, there are situations in which the perception system cannot accurately count all strawberries. The error rate is mainly due to the occlusion. Sometimes a single strawberry is divided into two due to stems (see highlight area Fig. 12(a)). Besides, the perception system cannot always recognize the overlapped strawberries (Fig. 12(b)). In real-world environments, some maturate strawberries are surrounded by stems and immature strawberries, to describe this type of situation more specifically, a cluster complexity is defined. That means if there are no obstacles surrounding the target strawberry, we classify this strawberry as easy to harvest, otherwise, it might be hard to harvest by the robot. In this paper, the robot focus on harvesting the strawberries with a low cluster complexity level.

### Analysis of ANN-based action system

**Fig. 14:**
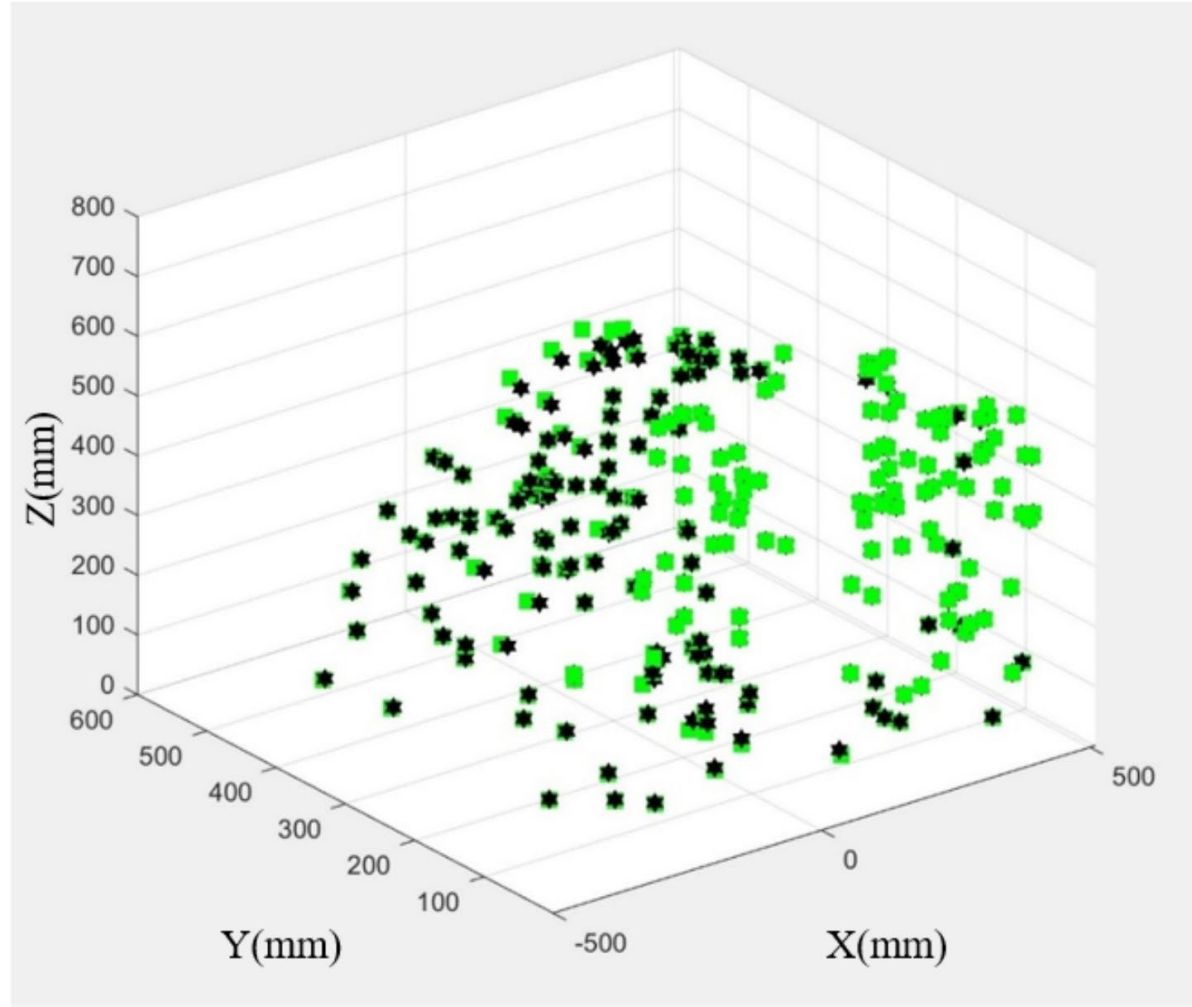
Target reaching accuracy for 200 points in the workspace

The robot manipulation action system is based on the neural control framework for goal-directed reaching. Once the 3D information of maturate strawberries is obtained, the goal has been decided. An example of results when PMP is given a target to reach is shown below. Figure 13(a) shows the transition from the initial position to the end-effector’s final target position. Similarly, Fig. 13(b) shows the sequence of arm joint angles in all DoF from its initial position to its final position for the end-effector to reach the target. The results are as expected, within a few millimetres of the target set. An important observation is the smoothness of the curves in the figures showing the framework’s natural no jerk feature. Finally, in Fig. 13(c), the graph shows the system’s time pressure to finish arm movement in the set number of iterations (1000). Figure 14 illustrates the actual target points (black) compared to the PMP solutions (green) with a mean error of 2.8853 mm. Note that some black dots are not visible as green dots cover them.

**Fig. 15:**
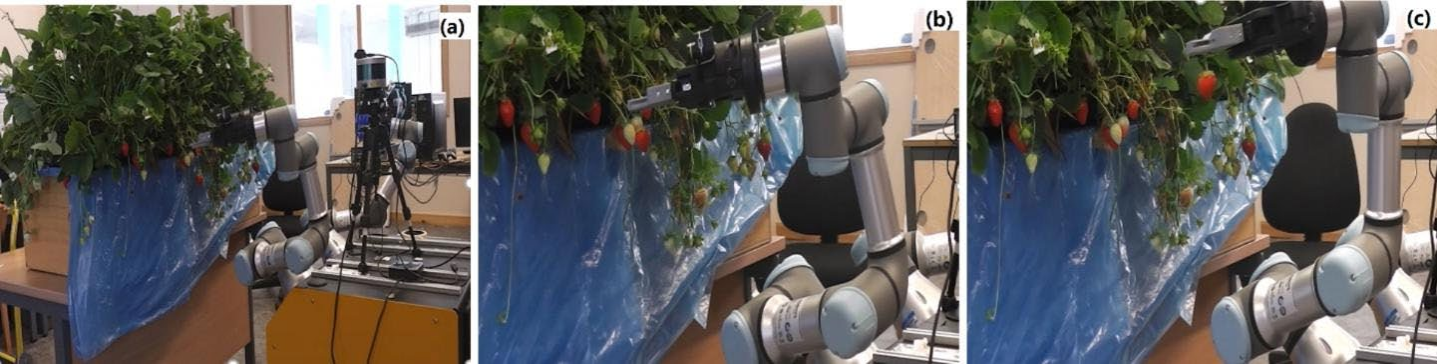
Test of the action system in a Lab setting: (**a**) the arm reaches the target position; (**b**) the gripper has cut the stem of the target strawberry; (**c**) the gripper remains closed until it goes to the specified position

Figure 15 shows the harvest process of strawberries. The robot first cuts the strawberry stem through its gripper to avoid touching the strawberries, and then the gripper remains closed until it moves to the specified position (punnet). More details of the laboratory and field trials may be found in supplementary files. Overall, these results indicate that the proposed perception-action system’s performance is effective and accurate, and the system can be smoothly applied to the actual robot platform.

### Field experiments and discussion

To show how the proposed robotic perception-action system works in real-world environments, the field experiments were carried out in the seasons 2020–2021 in the vertical greenhouse in Tiptree Essex, UK. The experiments were undertaken to test the whole system (perception, action, mobile base) with varying lighting conditions in the greenhouse. The harvesting process first obtains the 3D information of the target strawberry. Then the mobile base will determine whether it needs to move horizontally according to the distance to the strawberry. Finally, the action system calls the PMP to perform the harvesting. The 3D information keeps updating during the whole process to ensure the action system can always get the latest target’s coordinates.

However, according to our experiments, we noticed that the system could not distinguish between fully mature and soon mature strawberries. Especially those strawberries about to mature, more than 90% of their surface has been shown as red, and their ends may be slightly cyan. Therefore, we believe that the perception system can be combined with hyperspectral imaging technology to determine the maturity of strawberries in future work.

**Fig. 16:**
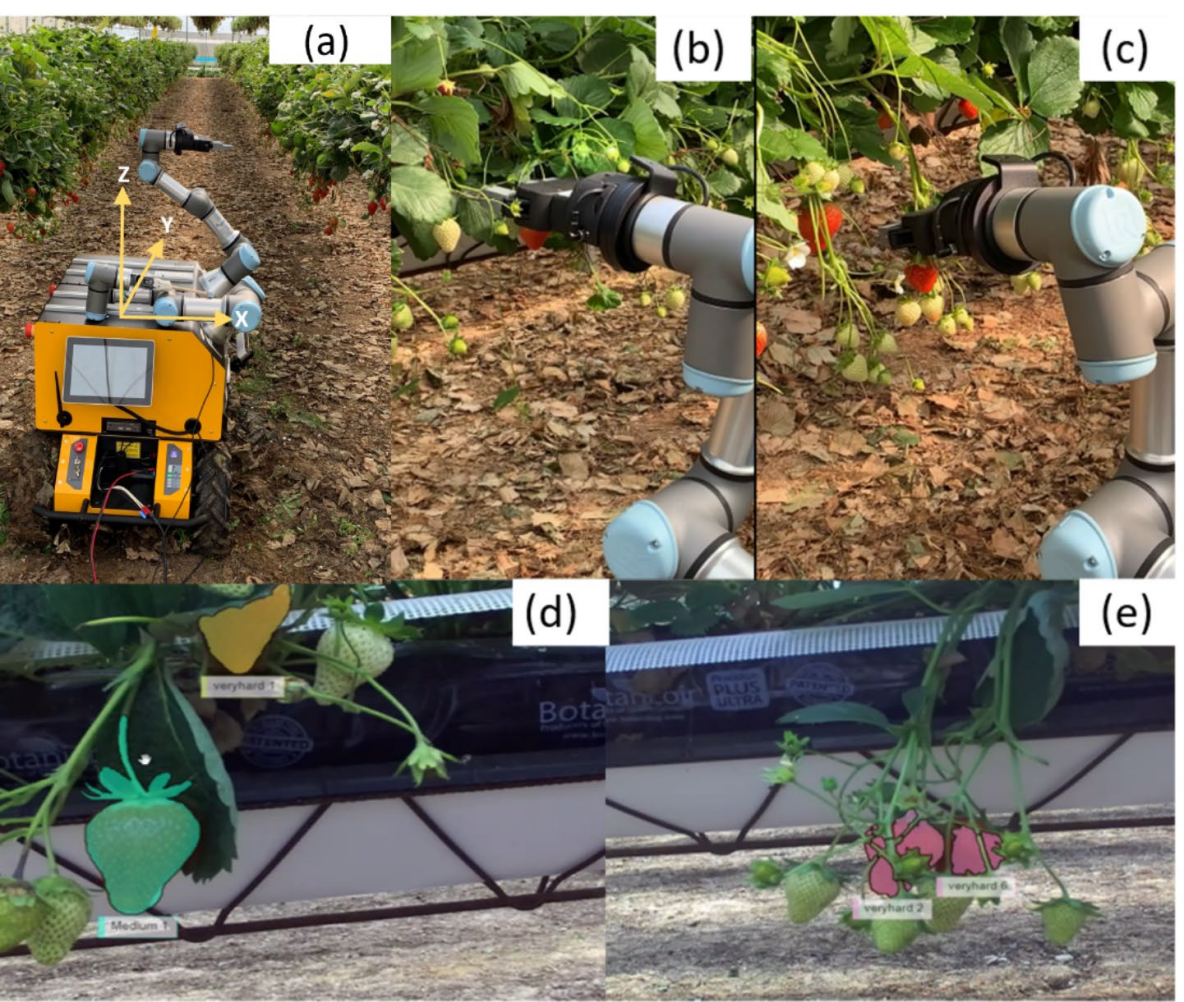
(**a**): The Essex robot is working in the greenhouse; (**b**): the robot is picking one ripe strawberry; (**c**): the robot is picking the strawberry which is surrounded by unripe strawberries; (**d**): a scenery with medium cluster complexity; (**e**): a scenery with high cluster complexity

Further, to test the perception-action system, whether the target strawberry is surrounded by obstacles (immature berries) and the stems are entangled with each other will affect the harvesting performance. As is shown in Fig. 16(b), there are no immature strawberries around for the target that are easy to reach and pick. As stated in the last section, the mean error of the action system is about 3 mm, which can guarantee the picking process is completed smoothly. However, there are some inevitable situations where obstacles surround the target strawberry (Fig. 16(c)). As mentioned before, cluster complexity is used to describe the situation. For example, Fig. 16(d) and Fig. 16(e) show two different cluster complexity levels. The gripper might simultaneously cut off mature and immature strawberries’ stems when the scenery has a high cluster complexity level. This situation is unacceptable for strawberry harvesting. Therefore, cluster complexity plays a vital role in harvesting performance. To improve the performance of our harvest robot, we believe it is necessary to introduce cluster complexity analysis and add alignment/fine-tuning operations in the action system.

Generally, because each cluster complexity is uncertain and random, it is still a strong challenge for the robot to realise the autonomous harvesting of the greenhouse. Besides, The harvesting efficiency also deserves further improvement. For example, as shown in Fig. 16, strawberries are located on both sides of the corridor, and the mechanical construction of the robot determines that it can only start picking from one side. Therefore, further work to improve the efficiency and harvesting performance in the complex cluster would increase the academic and commercial impact.

## Conclusion

This paper presented a biologically inspired ‘perception-action’ system for robotic soft fruit harvesting. The framework was field-tested in state of an art vertical growing system at Wilkin and Sons, Tiptree, Essex. Various core building blocks of the system are also configurable to other crops, like some initial results presented for tomato harvesting in greenhouses.

On the one hand, the proposed system uses conditional GAN trained on synthetically generated data which also incorporated a range of variance in lighting conditions and occlusions as observed in real-world conditions. The straightforward advantages of this are (1) elimination of the need for manual collection and labelling; (2) such kind of synthetic data can be generated for a range of other crops hence enabling configurability. According to the experimental results, the detection was reasonably robust for the perception system.

**Fig. 17:**
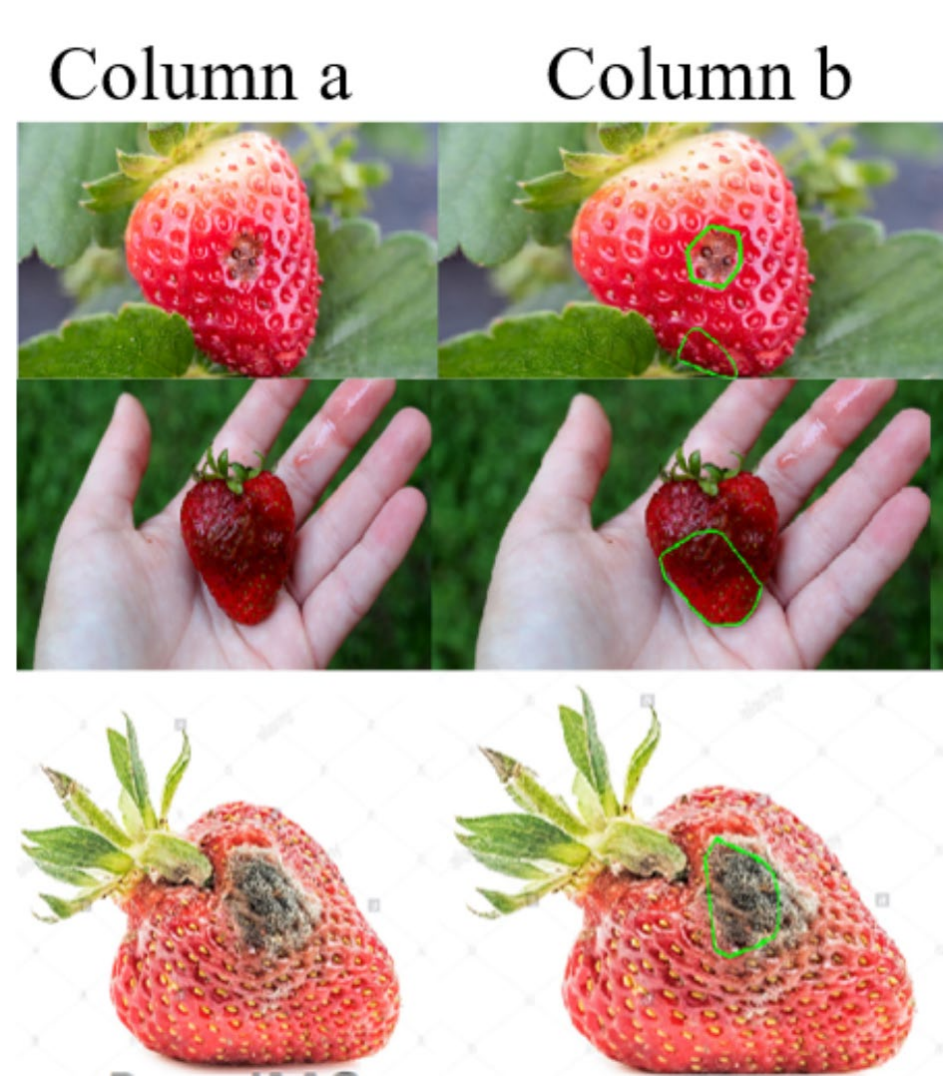
(**a**): Original images; (**b**) results of the rotten strawberry recognition model

The action system, on the other hand, was a Passive Motion Paradigm for goal-directed reaching and has a mean error of less than 3 mm. This paper first developed the neural control movement into the harvesting robots, which is a forward/reverse model that can be used to simulate the consequences of predictive planning and to extend a series of tools coupled with the arm. Compared with the traditional optimisation control method, this method can effectively solve the DoF problem and realise the high-precision movement of robotic arms. The results illustrated the overall performance of the action system and the smooth harvesting process. The architecture allows several future extensions:

1. *Configurability to other crops*. In greenhouses, some strawberries will become rotten. Therefore, identifying rotten strawberries is very meaningful for commercial farms. One of our ongoing works is trying to layer image datasets of healthy fruit with images of a similar shape and size as “rot”, then apply occlusion filters to these images to generate images of “rotting” fruit. This new data is generated by randomly placing occluded images of “rot” (which were other darker coloured objects and fruits) to simulate the presence of rot on a strawberry, and retraining the perception system, which serves to demonstrate the plausibility of using flexible hypothetical data for real-world situations - this approach can be taken not only for soft fruit but for a large variety of cross-industry applications. Figure 17 shows the initial results of the rotten strawberry recognition model.

2) *Strawberry Cluster Complexity Analysis and Bimanual Coordination.* Given the large variance in the structure of the canopy, cluster complexity analysis and bimanual coordination are our other ongoing works. In addition to the identification and localization of the berry, this features assigns a complexity level to every identified berry. This complexity level then enables planning of the strategy for picking like reaching with single-arm, arm and body movement and two-handed coordination (decluttering the obstacle with one hand and picking with the other one).

**Fig. 18:**
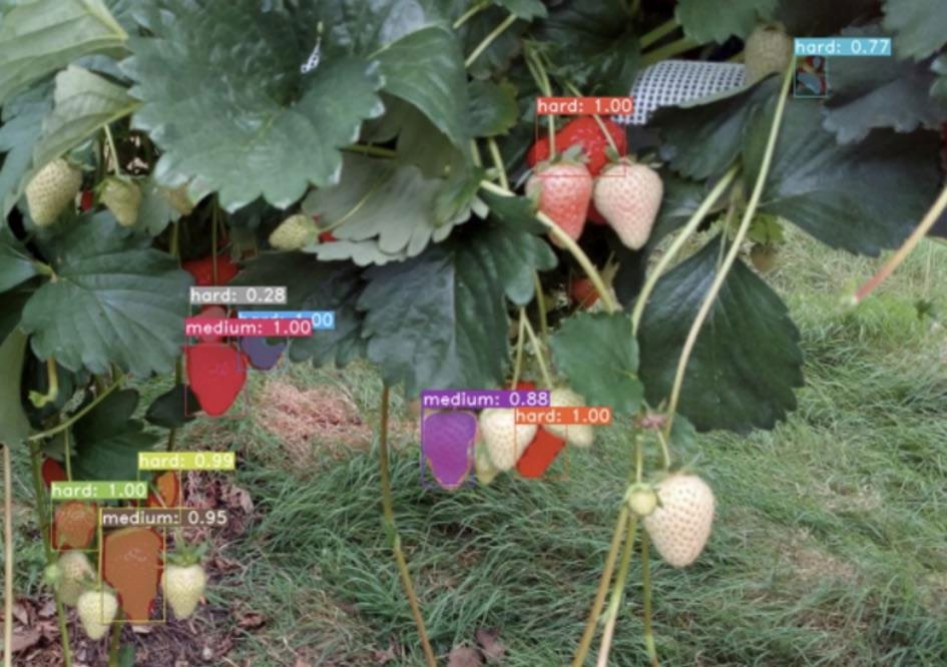
An example of the perception system with different levels of cluster complexity

As is shown in Fig. 18, there are some initial complexity analysis results of the perception system. Such analysis also enables predictive planning where with two cameras. One camera can cooperate with the robot to feedback on the target and gripper information in real-time in the ongoing working area. Another camera can identify the strawberries in the next picking-ready area and record the 2D/3D information and complexity level of each strawberry so that the picking sequence of the strawberries can be worked out, as well as the picking strategy of each strawberry.

3) *Other applications*. In the future, there are still some works that can be considered. For example, fruit counting and weight estimation are essential for crop phenotyping and yield analysis. These works can be realized by analysing point clouds. As is shown below, there is an example to collect the 2D and 3D data simultaneously from the farm by using the stereo camera. In future work, we aim to develop some point cloud analysis algorithms that will be used to deal with crop phenotype.

**Fig. 19:**
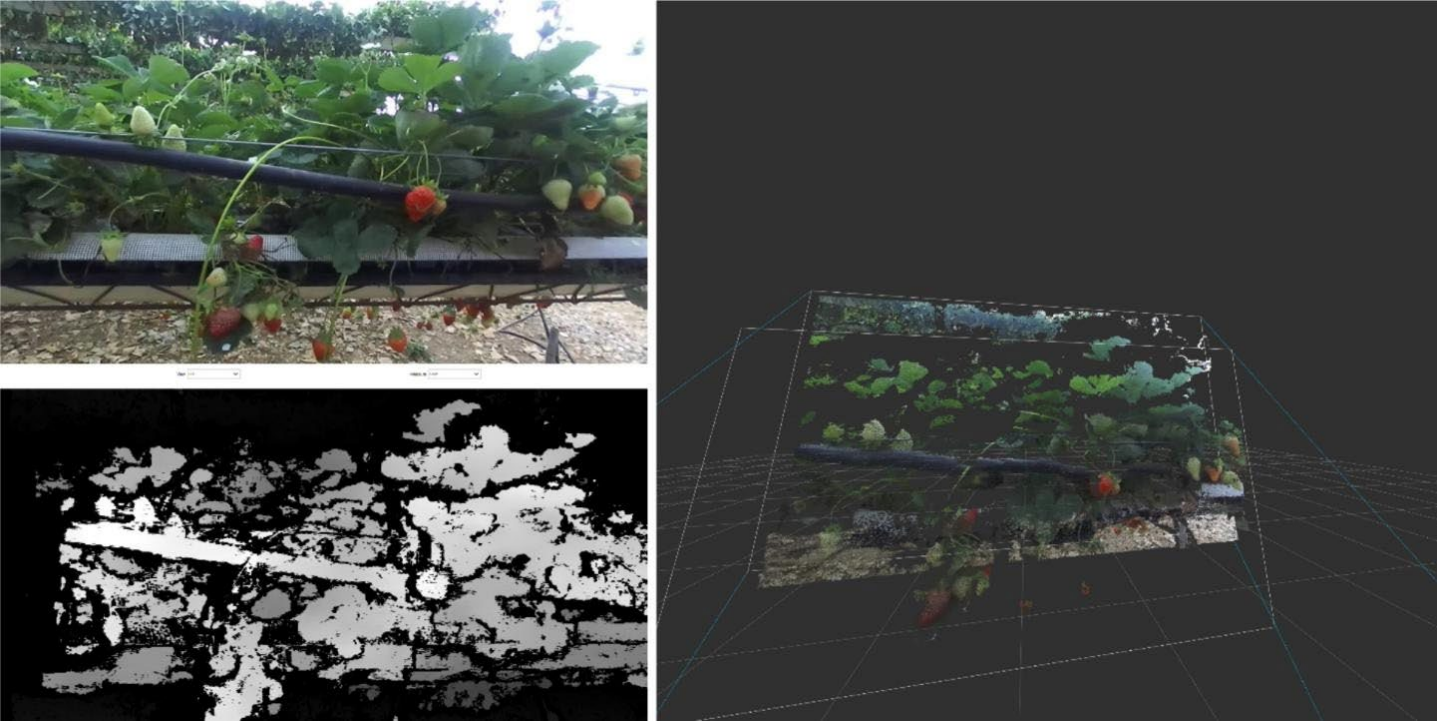
Obtaining 2D and 3D information for the dataset by using a stereo camera

Overall, the presented robot platform here has been applied to strawberries, which also has the distinct potential to be applied to other agriculture situations. Science robotics plays a key role in precision agriculture, and developing a more versatile harvesting system may be an important direction for agricultural robots.

## Electronic supplementary material

Below is the link to the electronic supplementary material.

Supplementary Material 1

## Data Availability

The data supporting the findings of this study’s perception system are openly available on GitHub: https://github.com/Fuli-Wang/synthetic-fake-data-sets.
